# Uncomplicated Amyand’s hernia in a setting of abdominal wall insufficiency: a case report

**DOI:** 10.1186/s13256-024-05015-y

**Published:** 2025-01-13

**Authors:** Vasishtha Avadhani Upadrasta, Avinash Koul, Vikrant Singh Chauhan

**Affiliations:** Dept. of General Surgery, Fortis Hospital, Sector 62, Noida, UP 201309 India

**Keywords:** Amyand’s hernia, Appendix, Hernia repair, Pantaloon hernia, Posterior wall deficiency

## Abstract

**Introduction:**

Amyand’s hernia, an uncommon condition characterized by the presence of the appendix within an inguinal hernial sac (< 1% incidence), poses diagnostic and therapeutic challenges. Often it is an intraoperative finding, with almost no clinical symptoms.

**Case presentation:**

This is a case of an Indian male in his early 80 years, diagnosed with bilateral direct inguinal hernias, one of which contained a noninflamed appendix. Given the thinned out abdominal wall, dense adhesions, and no demarcation between layers, the decision to proceed with a modified Bassini’s with Lichtenstein mesh repair without appendectomy, guided by intraoperative findings and the Losanoff–Basson Classification, reflecting the complex interplay between individual patient factors and intraoperative considerations. The patient did well during his postoperative stay and was in good health on a 45 day follow-up, with no complaints suggestive of recurrence or obstruction.

**Conclusion:**

This case underscores the importance of tailored management strategies and highlights, especially in cases where recurrence and postoperative wall integrity are in question, the ongoing need for research to refine treatment guidelines for Amyand's hernia, especially in cases of appendicitis not diagnosed preoperatively.

## Background

Hernia is defined as the protrusion or exit of any abdominal content(s), such as the small bowel, colon, omentum, bladder, uterus, or fallopian tubes, through a defect in the musculo-aponeurotic structures of the abdominal wall [1]. The lifetime incidence rate for abdominal hernias in Indian populations is given in Table 1 [2]. Indirect inguinal and femoral hernias, with contents presenting through respective canals, increase the risk of complications, such as obstruction, volvulus, and strangulation, proportionately.

**Table 1:** Prevalence of hernias according to types and the Losanof–Basson Classification

Type of hernia	Percentage
Right direct inguinal	20.93
Right indirect inguinal	22.50
Left direct inguinal	11.56
Left indirect inguinal	16.56
Bilateral inguinal	6.25
Umbilical	5.93
Paraumbilical	9.06
Epigastric	3.443
Incisional	4.12
Obturator	0.31
Traumatic	0.31

Losanoff–Basson classification			
Type	Features and content		Management
	Condition of appendix	Abdominal pathology (peritonitis, etc.)	
Type 1	Normal	Absent	Hernia reduction + mesh repair
Type 2	Acute appendicitis	Absent	Appendectomy only Primary closure without mesh repair
Type 3	Acute appendicitis	Abdominal wall and/or peritoneal sepsis	Laparotomy with appendectomy Primary closure without mesh repair
Type 4	Acute appendicitis	Other pathology	Managed as type 2 or 3, investigate pathology

The presence of an appendix within any hernia is quite a rare entity, amounting to no more than a 1% incidence rate. The first ever appendix in hernia was incidentally found by René Jacques Croissant de Garengeot in 1731, in a femoral hernia [3]. If the appendix presents as the content of an inguinal hernia, it is referred to as “Amyand’s Hernia,” named after Claudius Amyand, who found an appendix (perforated) in an incarcerated inguinal hernia of an 11-year-old boy, in 1735.

The presence of an appendix in an inguinal hernia amount to < 1% and the probability of it being inflamed (appendicitis) is 0.07–0.13%. The perforated appendix is seen in < 0.01% of cases with mortality of 15–30% due to severe sepsis [4–8].

Choosing a correct surgical treatment plan, which is predominantly made intraoperatively, is the single most crucial step in the successful outcome for the patient. Factors dictating the management of Amyand’s hernia are as per the Losanoff–Basson Classification (Table 1)[9].

## Case report

This is a case report regarding an Indian gentleman in his early 80 years, presenting with a complaint of swelling in bilateral groin regions for the past 7 months. The right side swelling had dull aching pain for the past 1 month, but no changes in bowel or bladder functions were noted. The swellings gradually increased in size over the said period, with no changes in the overlying skin color or texture. There was no history of fever or loose stools. There was no history of surgical intervention in the adjecent regions or the abdomen. The patient was a known case of poorly controlled hypertension and an asthmatic.

On physical examination, the swellings were both reducible, and the patient reported tenderness of right side on Valsalva. There was no focal tenderness, and bowel sounds were audible in the abdomen and both hernia sacs. The cough impulse was positive bilaterally, and the impulse was felt medially and not over the deep inguinal ring. A Deep Inguinal Ring (DIR) occlusion test was negative bilaterally. No evidence of hernia protruding into the scrotal sac was seen. Bilateral testicles were normal on physical examination. Sensory exam of the groin and perineum was normal. Preoperative blood work results were within normal limits. Ultrasonography of the abdomen confirmed the presence of bilateral direct inguinal hernias with free bowel movement, on the Valsalva maneuver. Due to the mobile nature of bowel loops, the presence of the appendix as part of hernia was not discerned. The right hernia sac showed significant peritoneal fluid collection. Given the comorbidities, the risks of postoperative ventilatory and cardiovascular complications were higher with general anesthesia (as compared with spinal anesthesia), and by recommendation of anesthesia team and with appropriate informed consent, the patient underwent bilateral open inguinal hernioplasty, under spinal anesthesia.

**Fig. 1:**
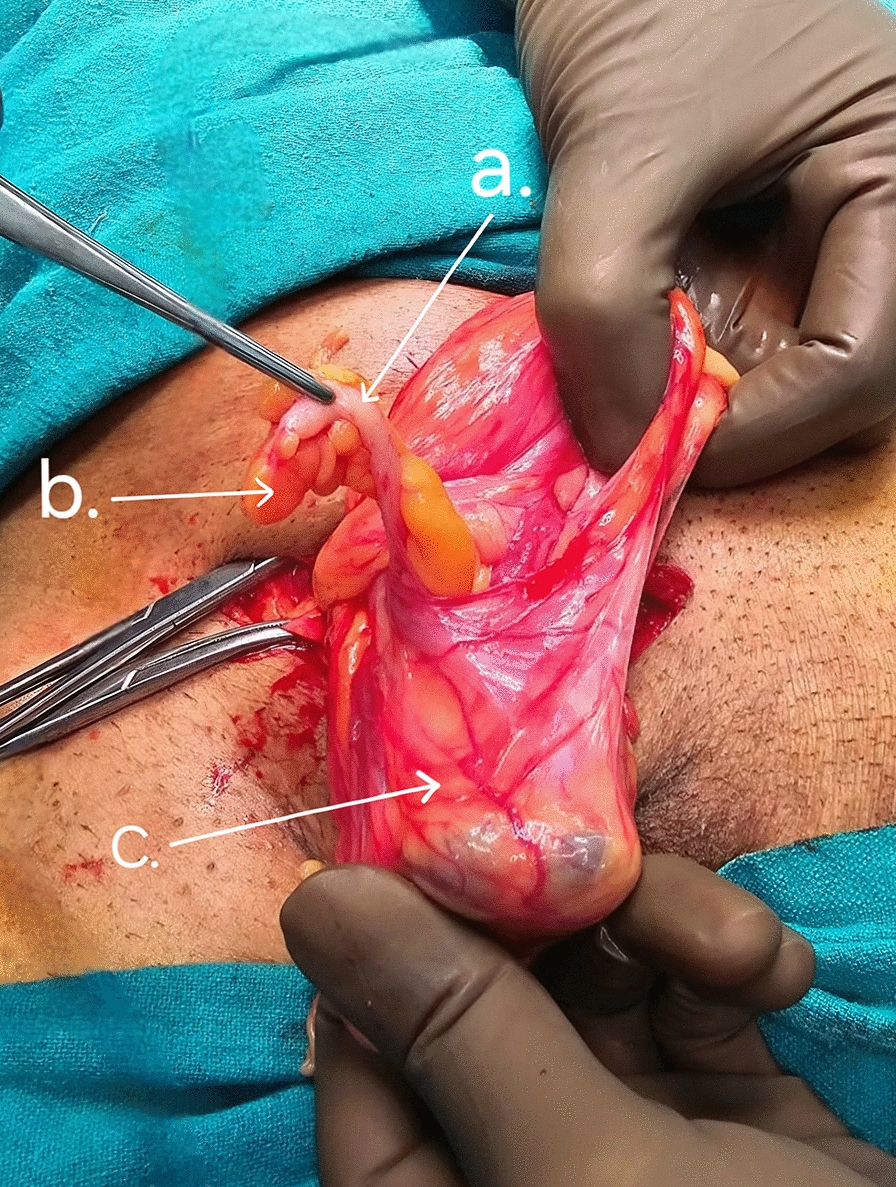
Vermiform appendix (**a**) along with its mesoappendix (**b**) seen within the right-sided direct inguinal hernia sac (**c**)

Intraoperatively, the right-side hernia was operated on first, revealing thinned-out abdominal muscles and aponeurotic fibers, dense adhesions between the layers of the abdominal wall with minimal differentiation seen between the fascia and the musculature. It was designated to be a direct inguinal hernia. The spermatic cord was identified and isolated. The sac was not separable (at the neck), medially, from the abdominal wall layers due to adhesions to the hernia defect, muscles, and aponeuroses. The distal lateral (retroperitoneal) part of cecum was noted as a sliding component of the hernia. The sac was excised at the neck and peritoneal fluid drained. Contents of the hernia sac were the distal ileum, cecum, vermiform appendix (noninflamed), and their respective mesentery. (Fig. 1).

The contents of the hernia were reduced into the abdominal cavity with ease, and the peritoneal sac was closed at the excised portion. Given that all three muscle layers and their aponeuroses were densely adherent to each other and they could not be individually separated without compromising further on their integrity, a modified Bassini’s repair with modified Lichtenstein repair using a nonabsorbable mesh was done. The polypropylene mesh was placed beneath what would be the transversalis fascia, and the deep ring was reconstructed. The combined musculoaponeurotic layers were fixed to the mesh and then approximated over it using nonabsorbable sutures. Prophylactic gentamicin irrigation of the surgical field containing the mesh was done, followed by layered abdominal wall closure. The rest of the surgery was uneventful. The patient was discharged the following day in good health and was accepting solid food orally and passing flatus and stools. His pain and discomfort levels were within expected limits throughout the period of stay.

The patient was advised to refrain from abdominal exercises, straining for defecation, and lifting heavy weights. He was advised for a high fiber diet and high protein diet for quicker healing of the wound. Appropriate physiotherapy and breathing exercises were discussed and adopted as required. He came for a follow-up visit after 10 and 45 days, with no complaints, a clean suture line, the ability to perform his daily tasks with ease, and no bowel or bladder dysfunction.

## Discussion

Amyand’s hernia is most frequently reported in men and is almost always seen on the right side. Exceptions to this are seen in situs inversus, malrotation, long appendix, or a loose cecum [4, 7, 10]. Seldom, it may be accompanied by cecum, bladder, ovary or fallopian tubes (females), or Mekel’s diverticulum. [11, 12]. Appendicitis, along with Amyand’s hernia, is a rare entity, with triggers usually being obstruction or direct trauma [4, 8, 10]. The identification and management of the type of Amyand’s hernia is as per the Losanoff–Basson calcification given below.

Preoperative diagnosis is rare, and intraoperative incidental finding is the most common level of identification of the entity. Velez *et al.* suggest that the treatment of choice if possible is via laparoscopic reduction of hernia. However, in cases where laparoscopy is not possible/available and the appendix is not pathological, Bassini’s repair or tension-free mesh repair is indicated. [13]. In case of an inflamed and/or perforated appendix, prosthetic mesh is avoided to reduce the risk of mesh contamination. [4]. It is speculated that, even in the case of uncomplicated acute appendicitis (type 2), the risk of using a synthetic mesh may outweigh the risk of recurrence following primary repair [13–15]. However, the advent of biological meshes has brought about a change in the consensus, but research and data on its outcome are still limited. The use of biological meshes is especially a viable alternative in patients of type 2 and 3 Amyand’s hernia, whenever a primary repair of the defect is not possible. [16, 17]. Given the fact that the appendix in the case was not inflamed, it was chosen to not undertake appendicectomy as it prevented further post operative morbidity. Also, the risk of exposure to bowel content (however miniscule) was avoided, allowing the safe placement of mesh (which was essential in this case due to abdominal wall insufficiency) without infection risk.

Another important aspect of the controversy is regarding the management in the case of an adherent and/or incarcerated appendix without inflammatory signs (no e/o appendicitis). Since hernia cannot be reduced before adhesiolysis (usually seen at the terminus of the appendix or base), the management is usually appendectomy, which may or may not be followed by a tension-free mesh repair. Kose *et al.* suggest that the risk of mesh infection in such cases is seldom [18]. In cases of recurrent hernias or previous surgeries in the local region, the management with tension-free mesh repair is statistically better performing than primary repair irrespective of the Losanoff–Basson type of hernia. Similarly, cases of acute appendicitis, both with [13] and without [14] perforation/exposure of bowel contents intraoperatively, seem to have normal outcomes even with mesh repairs. However, these are isolated cases and do not possess statistical significance due to paucity of research. Abdominal wall insufficiency cases with Amyand’s hernia are rare in literature, thus not giving us statistically significant directives to approach this case, whether or not they are based on the Losanof–Basson classification.

## Conclusion

The case reported herein is uncomplicated, with no evidence of appendicitis or appendicular adhesions. Hence it falls into the type 1 Amyand’s hernia according to the Losanoff–Basson classification. The management is a simple reduction of hernia contents and tension-free closure of hernia defect with mesh repair. Given that our patient’s abdominal wall was inherently weak, a decision to supplement the mesh repair with modified Bassini’s repair, with the closure of the floor of the hernia with polypropylene sutures, was made. However, it is to be noted that the Losanoff–Basson classification does not separate the management of hernias based on abdominal wall strength and, thus, might not be a comprehensive classification system for management choice in such cases. This case may be one of the rarer varieties of the Amyand’s hernia due to its presentation with abdominal wall insufficiency. This case report shall add to the current by delineating considerations to be made for use of mesh and the prerequisite steps to be taken when cases of appendices that may be infected and may have to be resected or the risks of not resecting have to be balanced against the need for a tension free mesh repair.

Associated literature was reviewed, and the lack in knowledge was identified in the following areas:

1. Should the management change in case of abdominal wall weakness/insufficiency?
2. When can a mesh repair be the management of choice, in type 2–4 Amyand’s hernia?
3. Is the Losanoff–Basson classification adequate for the choice of management? If so, which type does the incarcerated/adherent appendix, without appendicitis, fall into?

## Data Availability

No additional data to disclose.
